# Development and psychometric properties of a self-care behaviors scale (SCBS) among patients with rheumatoid arthritis

**DOI:** 10.1186/s41927-019-0069-4

**Published:** 2019-06-18

**Authors:** Haidar Nadrian, Yasna Hosseini Niaz, Zahra Basiri, Ahmad Tahamoli Roudsari

**Affiliations:** 1 Social Determinants of Health Research Center, Tabriz University of Medical Sciences, Tabriz, Iran; 2 Department of Health Education and Promotion, Faculty of Health, Tabriz University of Medical Sciences, Tabriz, Iran; 3 Department of Internal Diseases Medicine, Faculty of Medicine, Hamadan University of Medical Sciences, Hamadan, Iran

**Keywords:** Rheumatoid arthritis, Self-care behaviors, Instrumentation, Validity, Reliability

## Abstract

**Background:**

The role of self-care behaviors in promoting physical function, pain management, health status and quality of life among patients with Rheumatoid Arthritis (RA) is well documented. However, there is no valid and reliable instrument in the literature to assess such behaviors among the patients. In the present study, we aimed to develop and assess the psychometric properties of a Self-care Behaviors Scale (SCBS) among patients with RA.

**Methods:**

In 2017, applying a cross-sectional design, we recruited a convenient sample of 436 RA patients in Hamadan, Iran, to participate in the study. We developed the initial scale, including 30 items, after literature review, and having recommendations from an expert panel. Face, content, construct and convergent validity, as well as reliability of the scale were investigated.

**Results:**

In Exploratory Factor Analysis, the optimal solution comprising 25 items and 7 factors was emerged, which explained 62.5% of all variances between the items. In Confirmatory Factor Analysis, the measurement model fit the data well, and all subscales were significant within an acceptable range (χ2 [233] = 428.654, *p* < 0.0001, comparative fit index = 0.942, normed fit index =0.907, Tucker-Lewis index =0.916, and root mean square error of approximation = 0.043[(0.037–0.05]).

**Conclusion:**

The Self-care Behaviors Scale was found with appropriate validity, reliability, functionality and simplicity. To our knowledge, this scale is the only valid and reliable RA specific self-care behavior scale in the literature. Healthcare providers and health practitioners may apply the English version of this suitable instrument to find more valid and reliable data on RA self-care behaviors during primary assessments of the behaviors in educational interventions for the patients.

**Electronic supplementary material:**

The online version of this article (10.1186/s41927-019-0069-4) contains supplementary material, which is available to authorized users.

## Background

Rheumatoid arthritis (RA) is a chronic autoimmune disease that begins with periods of inflammation in synovium, and causes edema, swelling, vulnerability and stiffness in the joints [1–3]. It could be a major cause of disability, morbidity and mortality [2]. In addition to pain, motor limitation and disability, this disease leads to depression and anxiety, and extends its impacts to familial relationships of the patients [2, 4]. In patients with RA, the feeling of incompetence grows following the disability to perform former responsibilities [2, 5]. In the most of patients, there is deformation and reduced function of limbs, due to the involvement of moving joints [2]. About 1% of the world population suffers from RA [2, 6], and women are three times more likely to be diagnosed with the disease, compared to men [2, 7, 8] .The prevalence of disease has increased in recent years [9], and its rate varies from one group to another [2, 6]. On average, life expectancy among these patients is 3 to 7 years less than those of other people [2, 10].

In the United States, more than 2 million people are suffered from the disease and the current incidence is between 2 and 4 per 100,000 people, which is estimated to be more than 18% of the total population by 2020 due to the increased longevity of the population [7, 9]. In the Iranian studies conducted by Davatchi et al., the prevalence of RA is reported to be about 1% of the total population, which should be expected to increase in the upcoming years, due to the projected increase in the population of older adults in Iran [11, 12]. The major physical changes in these patients is associated with a risk of poor self-care [13], and thus poor quality of life [14].

As defined by WHO, “self-care” is “the ability of individuals, families and communities to promote health, prevent disease, maintain health, and to cope with illness and disability with or without the support of a health-care provider” [15]. The term self-care incorporates a wide scope from health promotion and disease prevention and control to providing care to dependent persons, and rehabilitation. According to Orem’s theory (1954), self-care is a regulated function of individuals based on the ability to carry out self-care behaviors on their own [16]. Self-care is also defined as a strategy to adapt to life events and tensions, which results in promoting healthy aging and independency. It also includes special activities that relieve the symptoms of illness and maintain and improve patients’ health [17–19]. A key factor in successful management of RA is the involvement of patients for correct self-care behaviors [1]. High levels of performing self-care behaviors (like fatigue management and energy conservation strategies, pain management, medication, exercise, nutrition and joint protection) may improve physical function, health status and quality of life (QOL) among RA patients [20–22]. Also, in order to promote self-care behaviors, these patients should be provided with a high level of practical, social, emotional and informational support [23].

Several previous studies have emphasized the role of self-care behaviors in promoting physical function, pain management, health status and QOL among patients with RA [24, 25]. In a previous study in Iran, the relationships between self-care behaviors, health status and QOL among patients with RA were investigated. In the study, a significant positive association was found between self-care behaviors and health status, but not between the behaviors and QOL. After more detailed analysis, the authors found that self-care may be indirectly associated to QOL through health status [21]. The diagnosis and provision of self-care needs of the patients are also asserted in the literature [24, 25]. Despite all abovementioned studies that have emphasized self-care behaviors among RA patients, the number of valid and reliable instruments to assess these behaviors is scarce. In 2011, Morowatisharifabad et al. developed and applied an invalidated self-care behavior scale to investigate these behaviors among the patients [26].

To our knowledge, there is no valid and reliable instrument in the literature to assess such behaviors among RA patients. In the studies conducted to investigate RA self-care behaviors, non-validated researcher-made questionnaires have been applied, which may potentially threat internal validity of the results. In order to address the self-care needs of RA patients, there is a great need to validated self-care behavior scales with the hope to provide health practitioners and nurses with valid and reliable data while designing health education and health promotion programs for the patients. Such instruments may also be useful while evaluating self-care education programs and services delivered to the patients at various levels of health systems. In the present study, we aimed to develop and assess the psychometric properties of a Self-care Behaviors Scale (SCBS) among patients with RA in Hamadan, Iran.

## Methods

### Participants and data collection

In this cross-sectional study, we recruited a convenient sample of 450 RA patients referring to a rheumatology clinic in Hamadan, Iran, to participate in the study. This nine-month study was conducted from May 2017 to February 2018. We estimated sample size based on 15 samples per item [27]. The primary version of scale comprised 30 items, and thus, a sample of 450 participants was considered as respondents. Inclusion criteria were (1) having at least four diagnostic criteria of RA, as suggested by American Rheumatology Association, (2) suffering from RA for more than 6 months, (3) being with at least 18 years of age and, (4) having no psychological or audio-visual problems. We recruited patients who fulfilled the inclusion criteria, consecutively, until the planned sample size was reached. Fourteen patients rejected to participate in our study (Response Rate = 96.8%). So, we included the data on 436 patients into analysis. We explained the purpose of study, as well as the patients’ rights as human subjects for the research to the participants. All those who accepted participation signed consent forms. In order to collect data, the first author conducted face-to-face private interviews with all participants in a private room at the clinic. The mean time to complete interviews was about 30–35 min.

### Measures

**Self-Care Behaviors Scale** developed by Morowatisharifabad et al. [26] was considered as the basis to develop the SCBS questionnaire. This scale included 17 items, within which the participants were requested to state “the frequency of performing various self-care activities for their arthritis on a regular basis (once a month) during the previous 12 months” [26]. A five-point Liker-type scaling ranged from zero (not at all) to four (always) was considered as the response format. The theoretical range for the scale was from zero to 68, within which the higher scores represented higher levels of performance in self-care behaviors.

**Arthritis Self-Efficacy Scale (ASES)** [28], the Persian version [26], was used to assess convergent validity of the SCBS. This scale comprises nine function items, five coping with pain items, and six items related to other RA symptoms (e.g. fatigue, depression). Due to logistical limitations, we chose to include the pain and other symptoms scale scores in the questionnaire, only. Response format was based on a four-point Likert-type scale: zero = not at all, one = seldom, two = sometimes, and three = a lot. The total score was in a range from 0 to 33, in which the higher scores indicated the higher levels of perceived self-efficacy among the patients.

**Demographic Data Form** was a nine-item scale developed by the researchers to collect data on socio-demographic characteristics of the respondents. The items included age, gender, occupation, marital status, level of education, household monthly income, residency place, disease duration and the history of RA in the family.

### Content validity

The initial 17-item scale was reviewed and assessed by an expert panel consisting two rheumatologists, a sports medicine specialist, an internal specialist with field experience in RA, a psychologist, two scholars in the area of health behavior and education and two nurses with field experience in RA. Based on the primary idea of the experts, the scale was not comprehensive in terms of assessing all domains of self-care behaviors. They finally recommended us to extend the scale through conducting a fast literature review on new relevant studies. So, we conducted a literature review. Based on the search results [22, 23, 29–34], 13 items were found to be added to the initial 17 items. Therefore, the first draft of the SCBS comprising 30 items was developed. In a second occasion, the draft was presented to the expert panel. During panel, the items were reviewed and assessed, orally, and evaluated in terms of appropriateness and relevance of items to RA patients, and response format, as well.

Content Validity Index (CVI) and Content Validity Ratio (CVR) were applied to validate the content of scale, quantitatively. Eleven specialists in the areas of health education and health behavior, rheumatology, sports medicine, psychology and nursing were requested to apprise the necessity of each item on the basis of a 3-point Likert-type scale (it is necessary, it is useful but not necessary, it is not necessary) (CVR). The values more than 0.99 (based on the Lawshe table) for each item was considered as necessary for the scale. The eleven experts were also asked to assess clarity, relevancy, and simplicity of the items, on the basis of a 4-point Likert-type scale. For each item, we considered the CVI value greater than 0.79 as appropriate and acceptable. So, the items with the score less than 0.79 were deleted from the scale.

We interviewed the panel of experts face-to-face to assess the items’ level of difficulty. We asked them to report the level of importance for each item, using a 5-point Likert-type scale (not important at all, a little important, moderately important, important, and absolutely important). Then, we calculated the impact score of the items through multiplying the frequency of an item by its mean importance [impact score = frequency (%) × importance]. Eventually, the items with impact score ≥ 1.5 were considered for the next stage.

The final draft was, then, pilot tested among a sample of 41 RA patients. In the pilot study, we aimed to assess the utility of scale, to identify the benefits and problems associated to the design, and to estimate the internal consistency of the scale, using Cronbach’s alpha coefficient. We did not include the pilot sample in the final sample.

### Translation into English

We translated the final version of SCBS into English, with the hope to be applied in future studies within different communities. As the SCBS was originally developed in Persian, we asked a native Persian speaker with mastery of the English language to translate it into English. In order to preserve the denotation and connotation of the items, we then back-translated [35] the scale into Persian by a native English speaker with mastery of the Persian language. The latter translator had not seen the original Persian version of the scale. Next, we compared the back-translated copy to the original Persian scale to recognize incongruities.

### Statistics

We used the statistical Package for Social Sciences (SPSS) v. 22 for the purpose of data entry, manipulation and analysis. No item was found with missing data. We used measures of central tendency and variability to summarize and organize the data. We then performed Pearson’s Correlation Coefficient, EFA, CFA, and Internal Consistency Reliability tests. The level of significance was considered 0.05, a priori.

### Construct validity

Applying principal component factor analysis with varimax rotation, we performed Exploratory Factor Analysis (EFA) to assess construct validity and factor structure of the scale. We also used Confirmatory Factor Analysis (CFA) with the robust maximum likelihood to estimate model parameters.

In order to determine the factor structure of the scale, an EFA was conducted based on a randomized split of the data in the sample. We randomly selected a sample of 200 participants using the randomization function on SPSS v. 22. During EFA, we considered the factor loadings equal or greater than 0.3 to be appropriate, and the eigenvalues above 1 as an assignment for the number of factors. We then used the Kaiser-Meyer-Olkin (KMO) and Bartlett’s Test of sphericity to obtain the appropriateness of sample.

Thereafter, we performed a CFA on the remaining 236 participants of the larger overall sample to identify whether the factor structure required modification. The Analysis of Moment Structures (AMOS), version 10.0 was applied to conduct the CFA. In the CFA process, the absolute fit of the model to data was evaluated using the χ2 statistic, the comparative fit index (CFI), the Tucker–Lewis Index (TLI), and the root mean square error of approximation (RMSEA) tests. We considered the model to be acceptable if χ2 was between 1 and 5, CFI was more than 0.8, TLI was greater than 0.9, RMSEA was < 0.05 good fit or between 0.05 and 0.08 adequate fit.

### Reliability

We used Cronbach’s alpha test to investigate internal consistency of the scale. The Cronbach’s alpha coefficient of 0.7 or above was considered to be acceptable. We also applied Intra class Correlation Coefficients (ICC) to calculate the test-retest reliability coefficient (ICC ≥ 0.70 was considered satisfactory).

### Convergent validity

We applied Pearson’s correlation coefficient test to assess the nature of associations between the SCBS factors, and to evaluate the associations between the factors and the domains of ASES.

### Ethical considerations

Ethics committee in Tabriz University of Medical Sciences approved the study (number 40773, 16.11.2017). We obtained informed consent form from all respondents, and all signed consent forms. We also explained the patients about the purpose of study, and assured then on the confidentiality of their data.

## Results

### Participants

In this study, data on 436 patients with RA were analyzed. The age of participants ranged from 18 to 84 years (Mean = 53, SD = 13). A majority of participants were female (87%), married (79%), housewife (77%) and urban resident (68%). About 60% aged 50 years and older and 71% had primary or lower levels of education. Duration of RA among 81% of the patients was 3 years and higher.

### Content validity

Based on the qualitative recommendations of the experts and the quantitative results (CVI and CVR), 7 out of 30 items were revised. The qualitative recommendations of the expert panel, which was mostly regarding technical revisions, wording and phrasing of the items, was considered to revise and modify the instrument. Also, as they recommended, 3 items out of the initial 30 items of the instrument were removed, and therefore, the SCBS with 27 items was included in the CVR and CVI processes. In quantitative evaluation, CVI (ranged between 0.8 and 1) and CVR (ranged between 0.6 and 1) showed satisfactory results for each item, and consequently for the SCBS. The Impact Score and the CVR value for all SCBS items were more than 1.5 and 0.62 [36], respectively, and thus no item was deleted; however, in qualitative content validity, some modifications were made to the wording and phrasing of some items. Eventually, 27 items remained.

### Factor structure

In EFA, Kaiser-Meyer-Olkin measure of sampling adequacy for the SCBS was 0.804 (Approx. Chi-Square = 4811.324, df = 300, p < = 0.001). In the Communalities table, two items (items number 15 and 24) were found to be with extraction values less than 0.2. So, we removed these two items and rerun EFA. Table 1 shows the factors (subscales), number of items, range, mean and standard deviation, kurtosis and skewness, as well as floor and ceiling effects of the factors.

**Table 1:** A summary of characteristics of the factors

Factors (Subscales)	Number of items	Range	Mean (SD)	Kurtosis	Skewness	Floor effect (%)	Ceiling effect (%)
F1: Physical Activity	6	0–24	13.29 (2.9)	−.311	.117	0	0
F2: Medication	3	0–12	10.4 (2.4)	1.8	−1.53	.4	6.3
F3: Stress Management/Others	4	0–16	6.01 (2)	1.67	1.48	0	.2
F4: Nutrition/Joints Protection	4	0–16	4.19 (2.9)	.78	.89	4.9	.2
F5: Management of Daily Activities	3	0–12	6.48 (2.4)	−.36	−.009	.7	2.4
F6: Pain Management	3	0–12	3.89 (2.9)	−.38	.34	7.1	0
F7: Tobacco/Opium Use.	2	0–8	7.08 (1.6)	1.93	−1.44	.7	7.1

In the last iteration of analysis, seven distinctive factors were extracted. These factors explained 62.5% of total variance between the items. Cattell’s scree test also indicated the possibility to extract four to seven factors. Therefore, we conducted multiple runs of factor analysis with various numbers of factors. Finally, we distinguished the initial seven-factor solution as the most distinct pattern for factor loadings.

Rotated factor pattern coefficient for the solution is shown in Table 2. For each factor, we presented information about initial eigenvalues (before rotation), rotation sum of squares (variance accounted for after rotation), and percent of variance explained (after rotation). In the table, we also provided internal consistency reliability, as indicated by Cronbach’s alpha, and ICC with 95% confidence intervals (CI) for each factor.

**Table 2:** Rotated factor pattern coefficients for variable solutions (25 items) of SCBS factors

	In the past year, how often have you done regularly the following activities for your arthritis? (By ‘regularly’ we mean roughly once a month)							
	Factors^*^	F1^*^	F2	F3	F4	F5	F6	F7
Sc-1	Exercised (including water exercise)	.919						
Sc-6	Replaced higher-intense exercises with lower-intense options, in the case of having a mild pain after exercise	−.905						
Sc-5	Stopped exercise when having severe joint pains after exercise	−.891						
Sc-2	Exercised weekly with moderate intensity	.850						
Sc-4	Balanced between rest and exercise periods, if needed	−.841						
Sc-3	Exercised daily with moderate intensity	.792						
Sc-21	Changed the dosage of your drugs or the time of taking them without informing your physician		.893					
Sc-19	Taken your drugs regularly and based on your prescription		.882					
Sc-20	Visited your physician regularly		.814					
Sc-18	Used relaxation methods such as meditation			.777				
Sc-17	Used methods to help control stress			.637				
Sc-27	Used larger joints instead of smaller joints (e.g. pushing in a table by the hip joint instead of wrist joint)			.619				
Sc-26	Taken supplements containing fish oil or omega-3 without consulting your physician			−.468				
Sc-13	Avoided certain foods				.742			
Sc-14	Used massage				.620			
Sc-12	Taken food supplements, vitamins, or eaten special foods			.327	.508			
Sc-9	Used joint protection, bracing, or splinting				.496		.369	
Sc-10	Rested					.734		
Sc-11	Adjusted your daily routine or work schedule			.307		.660		
Sc-16	Talked with persons who are sympathetic					.610		
Sc-7	Used a heated pool, tub, or shower						.696	
Sc-8	Applied heat to parts of your body						.675	
Sc-25	Used some facilities (like handles, armchair and so on) in toilet, bed room and bathroom to ease the processes of sitting down, standing up and walking.						.389	
Sc-23	Used substances, like opium, to control pain.							.803
Sc-22	Smoked cigarette or hookah							.737
	*Initial Eigenvalues*	*5.51*	*2.68*	*2.56*	*1.36*	*1.24*	*1.18*	*1.07*
	*Rotation sums of squares*	*4.71*	*2.47*	*2.02*	*1.88*	*1.69*	*1.56*	*1.26*
	*Percent of variance explained*	*22.05*	*10.74*	*10.24*	*5.45*	*4.97*	*4.74*	*4.28*
	*Cronbach α*	*.533*	*.85*	*.66*	*.60*	*.67*	*.68*	*.29*
	*ICC (95% CI)*	*.75 (.72–.78)*	*.84 (.81–.88)*	*.86 (.82–.9)*	*.83 (.79–.86)*	*.81 (.78–.84)*	*.89 (.86–.92)*	*.91 (.89–.94)*

*F1 = Physical Activity; F2 = Medication; F3 = Stress Management/Others; F4 = Nutrition/Joints Protection; F5 = Management of Daily Activities; F6 = Pain Management; F7 = Tobacco/Opium Use; Extraction Method: Principal Component Analysis.; Rotation Method: Varimax with Kaiser Normalization

**Fig. 1:**
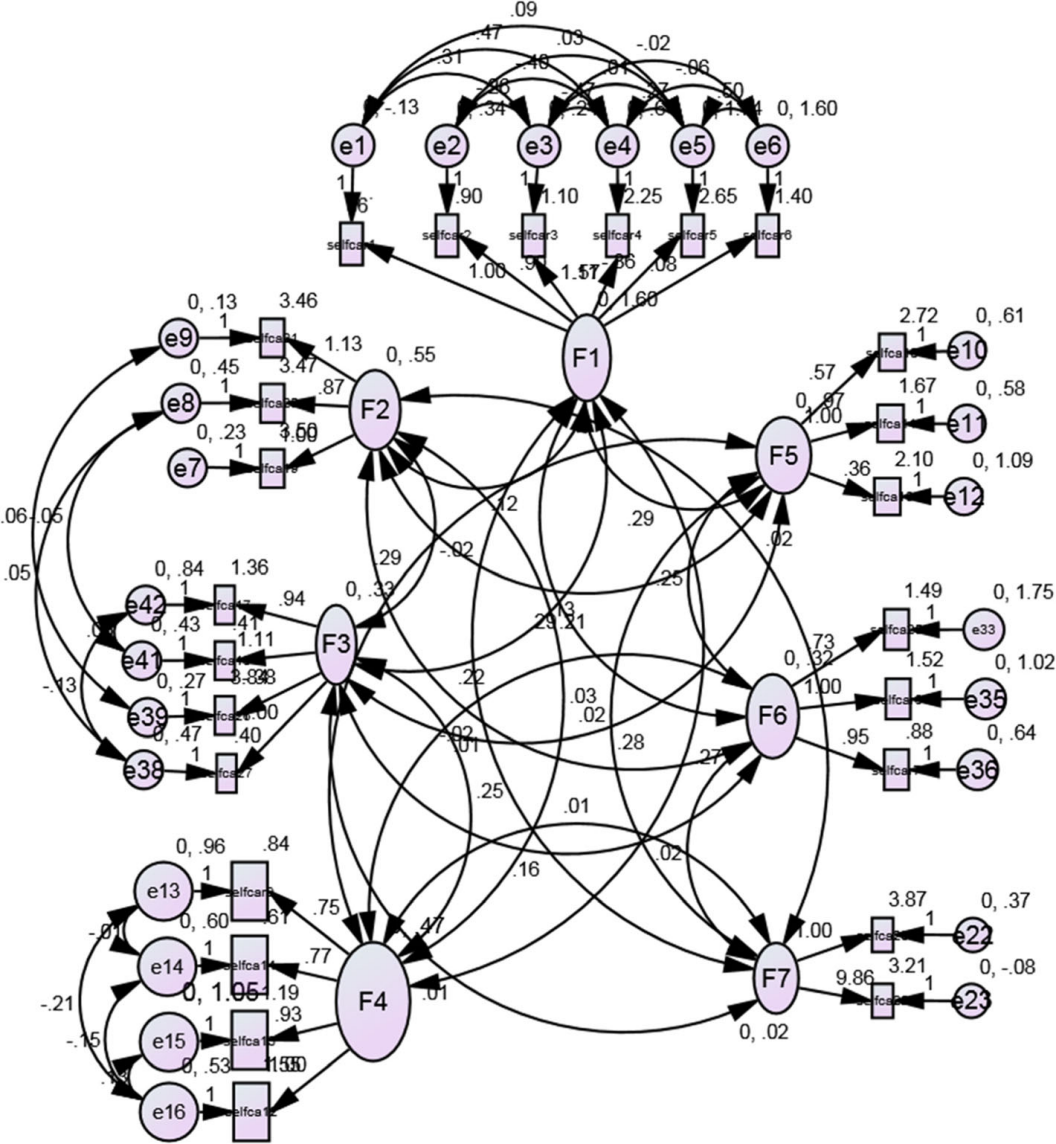
CFA based relations between the items and the factors and between the factors. All relations between the factors and items and between the factors were statistically significant (All *P* < 0.05). F1 = Physical Activity; F2 = Medication; F3 = Stress Management/Others; F4 = Nutrition/Joints Protection; F5 = Management of Daily Activities; F6 = Pain Management; F7 = Tobacco/Opium Use. Fit indices: χ2 [233] = 428.654, *p* < 0.0001, CFI = 0.942, NFI = 0.907, TLI = 0.916, RMSEA = 0.043[(0.037–0.05]

We found the measurement model (Fig. 1) to be with a good fit to data in the assumed model. All subscales were found to be significant within an acceptable range (χ2 [233] = 428.654, *p* < 0.0001, CFI = 0.942, NFI = 0.907, TLI = 0.916, RMSEA = 0.043[(0.037–0.05]).

### Reliability

The Cronbach’s alpha coefficient for the total scale, in the pilot and final data were 0.85 and 0.74, respectively. For the factors, a wide range from low to high levels of Cronbach’s alpha coefficients was found (Table 2). Factor 2 had the highest (0.85) and factor 7 had the lowest (0.29) Cronbach’s alpha. The Cronbach’s alpha for some of the factors (F1, F4, and F7) were less than 0.65, which argued omitting of the factors. We, therefore, considered visual inspection and hyperplane count [37] to determine the simple structure and the best solution, respectively. Finally, we decided not to eliminate the factors considering the importance of self-care domains presented in the factors. For all subscales, ICC with 95% CI was higher than .70 (.71–.91) (Table 2).

### Convergent validity

In the process of investigating convergent validity, we found significant correlations between a majority of the factors and the subscales of ASES (pain and other symptoms scales) (Table 3). Statistically significant relationships were also found between the most of factors after applying Pearson correlation coefficient test. The strongest relationships were found between factor 3 (stress management/others) and factor 5 (management of daily activities) (r = .356), and factor 4 (Nutrition/Joints Protection) and factor 6 (Pain Management) (r = 0.356). The weakest relationships was found between factor 1 (Physical Activity) and factor 7 (Tobacco/Opium Use) (r = −.006) (Table 4).

**Table 3:** Pearson correlation coefficients of the SCBS factors, and Arthritis Self-efficacy Scale components scores

Factors/Constructs	Arthritis Self-efficacy Scale		Total Self-efficacy
	Pain self-efficacy	Other symptoms self-efficacy scale	
F1: Physical Activity	.002	−.004	−.001
F2: Medication	.032	.106^a^	.080
F3: Stress Management/Others	.236^b^	.482^b^	.411^b^
F4: Nutrition/Joints Protection	−.017	.166^b^	.089
F5: Management of Daily Activities	.236^b^	.240^b^	.269^b^
F6: Pain Management	.195^b^	.154^b^	.142^b^
F7: Tobacco/Opium Use.	.041	.125^b^	.096^a^

^a^Correlation is significant at the level of 0.05 (2-tailed); ^b^Correlation is significant at the level of 0.01 (2-tailed)

**Table 4:** Pearson’s correlation coefficients between the factors

Factors	1	2	3	4	5	6	7
F1: Physical Activity	1						
F2: Medication	.123^b^	1					
F3: Stress Management/Others	.030	.041	1				
F4: Nutrition/Joints Protection	.079	.013	.316^b^	1			
F5: Management of Daily Activities	.040	.155^b^	.356^b^	.304^b^	1		
F6: Pain Management	.113^a^	.021	.220^b^	.356^b^	.300^b^	1	
F7: Tobacco/Opium Use.	−.006	.130^b^	.058	.024	.125^b^	.142^b^	1

^a^Correlation is significant at the level of 0.05 (2-tailed); +bCorrelation is significant at the level of 0.01 (2-tailed)

## Discussion

In the present study, we reported the development and psychometric properties of the SCBS (Additional file 1: Table S1) to assess self-care behaviors among patients with RA. Despite the importance of self-care behaviors among RA patients [22, 29, 30, 32–34], there is a scarcity in the valid and reliable instruments to be applied for investigating the behaviors during health promotion interventions among RA patients. Although the initial version of the scale was developed in a previous study [26], psychometric properties of the scale were still unclear. So, we decided to develop the instrument and test it for validity and reliability.

In construct validity, the seven-factor solution yielded a distinct pattern of factor loadings. This solution explained about 62.5% of total variance between the items. The first three factors, including “physical activity”, “medication”, and “stress management/others” were particularly so strong that together explained about 43.03% of the total variance. As confirmations for factor structure of the scale, the measurement model fit the data well and all factors were significantly in an acceptable range. Therefore, the construct validity of the instrument was approved.

As recommended by Gorsuch [38], we investigated correlations between the factors to assess convergent validity of the scale. In results, we found a wide range of various associations between the factors. The variations were from none to moderate and strong relationships, which may be due to the nature of factors. Some of the factors covered a wide range of self-care behaviors. As instances, the strongest and the weakest relationships were found between factor 3 (stress management/others) and factor 5 (management of daily activities), and between factor 1 (physical activity) and factor 7 (tobacco/opium use), respectively. Presenting associations between factors in our study may be helpful for future researchers in comparing their results with those found in the present study [39]. Such correlations between the factors may be interpreted like alpha indicating the stability of each factor [38].

Our results also showed that some factors derived from the SCBS had weak to moderate associations with the two components of ASES. A majority of the associations were found to be positive between almost all factors with both RA pain and other symptoms components of the ASES. In other words, the higher was the level of self-efficacy in different domains, the higher level of self-care behaviors among the patients was inferred. All these findings affirmed the convergent validity of the scale.

Our results also confirmed acceptable internal consistency of the SCBS. The Cronbach’s alpha coefficient for the scale was 0.74, which was at an acceptable range, as recommended by Sim and Wright [40] and Develis [41]. However, as we expected, Cronbach’s alpha for some of the factors (F1, F4, and F7) were less than 0.65, which argued omitting of the factors. After visual inspection and the hyperplane count [37], we finally decided not to eliminate the factors considering the importance and relevancy of the factors. We also expected low levels of Cronbach’s alpha for these factors. For factor 4, as an instance, low internal consistency may be related to different nature of the items loaded on the factor. The items regarding both nutrition and joint protection behaviors, as quite distinct self-care behaviors, were loaded on this factor. Based on Scree plot visual inspection, factor analysis was rerun for several times to find a clearer solution. However, the seven-factor solution was found as the best solution. As factor 4 in this solution included these two domains, the low Cronbach’s alpha was inevitable. For factor 7, low level of Cronbach’s alpha may be attributed to the few item numbers loaded on the factor. As another indication for internal consistency of the SCBS, test-retest reliability for all subscales was higher than .70 (.71–.91). In several previous studies [39, 42–44], Cronbach’s alpha and/or ICC have been used to confirm the reliability of instruments. Moreover, the results of CVI and CVR as well as those of face and content validity in the present study ensured the simplicity, clarity and relevancy of the SCBS.

A limitation for our study was the difficulty in comparing SCBS with other similar instruments, which was due to the lack of comparable standard instruments in the literature. At the end of study, we found that some items might be beneficial for some patients only (e.g. applying heat to body parts) and some items cover more than one object/issue for patients to assess (e.g. changed dosage of drugs or time of intake). For future studies, therefore, we suggest using a psychometric method that offers insights into item-based parameters, such as Rasch analysis. Another important further requirement of measurement which may be addressed in the Rasch model is invariance. This may help the future researchers to eliminate redundant items and to improve the formulation of some items.

## Conclusion

In our study, the SCBS was found to be with an appropriate level of validity, reliability, simplicity, and functionality. To the best of our knowledge, this scale is the only valid and reliable RA specific self-care behavior scale in the literature. Nurses, healthcare providers, health practitioners and RA researchers may use this suitable instrument to find more valid and reliable data on self-care behaviors during primary assessments of behaviors for RA educational interventions and health promotion programs. Further research applying this scale is suggested to compare various aspects of the SCBS in different RA populations.

## Additional file

Additional file 1: **Table S1.**. Self-Care Behaviors Scale (SCBS) developed in the present study. A copy of the Self-Care Behaviors Scale (SCBS) developed in the current study is presented here. The subscales, number of items for each subscale, internal consistency and test-retest reliabilities are presented in the paper. The box ticked for each item, is the score for the item. If two consecutive boxes are circled, code the lower number (less self-care). Do not score the item, if the boxes are not consecutive. The mean of the items is considered as the score for the scale. Do not score the scale, if more than 25% of the items are missing. The theoretical range for the scale was from zero to 100, within which the higher scores represent higher levels of performance in self-care behaviors. (DOCX 14 kb)

## Data Availability

The data that support the findings of this study are stored and available upon reasonable request.

## Abbreviations

ASES: Arthritis Self-efficacy Scale
CFA: Confirmatory Factor Analysis
CFI: Comparative fit index
CVI: Content Validity Index
CVR: Content Validity Ratio
EFA: Exploratory Factor Analysis
ICC: Intra class correlation coefficients
KMO: Kieser-Meyer-Olkin
QOL: Quality of life
RA: Rheumatoid Arthritis
RMSEA: Root mean square error of approximation
SCBS: Self-care Behaviors Scale
TLI: Tucker–Lewis Index
