# Supplementary material for: Development and psychometric properties of a self-care behaviors scale (SCBS) among patients with rheumatoid arthritis
Source: BMC Rheumatol. 2019 Jun 18;3:4. doi: 10.1186/s41927-019-0069-4 (PMC6582489; doi:10.1186/s41927-019-0069-4)
Supplement: Supplementary file 1 — Table S1.. Self-Care Behaviors Scale (SCBS) developed in the present study. A copy of the Self-Care Behaviors Scale (SCBS) developed in the current study is presented here. The subscales, number of items for each subscale, internal consistency and test-retest reliabilities are presented in the paper. The box ticked for each item, is the score for the item. If two consecutive boxes are circled, code the lower number (less self-care). Do not score the item, if the boxes are not consecutive. The mean of the items is considered as the score for the scale. Do not score the scale, if more than 25% of the items are missing. The theoretical range for the scale was from zero to 100, within which the higher scores represent higher levels of performance in self-care behaviors. (DOCX 14 kb) [file 41927_2019_69_MOESM1_ESM.docx]

|  | **In the past year, how often have you done regularly the following activities for your arthritis? (By ‘regularly’ we mean roughly once a month)** | Not at all | Rarely | Sometimes | Often | Always |
| --- | --- | --- | --- | --- | --- | --- |
| 1 | Exercised (including water exercise) |  |  |  |  |  |
| 2 | Replaced higher-intense exercises with lower-intense options, in the case of having a mild pain after exercise |  |  |  |  |  |
| 3 | Stopped exercise when having severe joint pains after exercise |  |  |  |  |  |
| 4 | Exercised weekly with moderate intensity |  |  |  |  |  |
| 5 | Balanced between rest and exercise periods, if needed |  |  |  |  |  |
| 6 | Exercised daily with moderate intensity |  |  |  |  |  |
| 7 | Changed the dosage of your drugs or the time of taking them without informing your physician |  |  |  |  |  |
| 8 | Taken your drugs regularly and based on your prescription |  |  |  |  |  |
| 9 | Visited your physician regularly |  |  |  |  |  |
| 10 | Used relaxation methods such as meditation |  |  |  |  |  |
| 11 | Used methods to help control stress |  |  |  |  |  |
| 12 | Used larger joints instead of smaller joints ( e.g. pushing in a table by the hip joint instead of wrist joint) |  |  |  |  |  |
| 13 | Taken supplements containing fish oil or omega-3 without consulting your physician |  |  |  |  |  |
| 14 | Avoided certain foods |  |  |  |  |  |
| 15 | Used massage |  |  |  |  |  |
| 16 | Taken food supplements, vitamins, or eaten special foods |  |  |  |  |  |
| 17 | Used joint protection, bracing, or splinting |  |  |  |  |  |
| 18 | Rested |  |  |  |  |  |
| 19 | Adjusted your daily routine or work schedule |  |  |  |  |  |
| 20 | Talked with persons who are sympathetic |  |  |  |  |  |
| 21 | Used a heated pool, tub, or shower |  |  |  |  |  |
| 22 | Applied heat to parts of your body |  |  |  |  |  |
| 23 | Used some facilities (like handles, armchair and so on) in toilet, bed room and bathroom to ease the processes of sitting down, standing up and walking. |  |  |  |  |  |
| 24 | Used substances, like opium, to control pain. |  |  |  |  |  |
| 25 | Smoked cigarette or hookah |  |  |  |  |  |

**Additional File:** Self-Care Behaviors Scale (SCBS) developed in the present study

**Scoring**

The subscales, number of items for each subscale, internal consistency and test-retest reliabilities are presented in the paper. The box ticked for each item, is the score for the item. If two consecutive boxes are circled, code the lower number (less self-care). Do not score the item, if the boxes are not consecutive. The mean of the items is considered as the score for the scale. Do not score the scale, if more than 25% of the items are missing. The theoretical range for the scale was from zero to 100, within which the higher scores represent higher levels of performance in self-care behaviors.
